# Proximate Analysis of Selected Macroalgal Species from the Persian Gulf as a Nutritional Resource

**DOI:** 10.21315/tlsr2020.31.1.1

**Published:** 2020-04-07

**Authors:** Kiana Pirian, Zahra Zarei Jeliani, Mitra Arman, Jelveh Sohrabipour, Morteza Yousefzadi

**Affiliations:** 1 Department of Biotechnology, Faculty of Agriculture, Buali-Sina University, Hamedan, Iran; 2 Department of Marine Biology, Faculty of Marine Science and Technology, University of Hormozgan, P.O.Box 3995, Bandar Abbas, Iran; 3 Department of Biology, Payame Noor University (PNU), P.O. Box 19395-3697, Tehran, Iran; 4 Natural Resources Department, Hormozgan Agricultural and Natural Resources Research and Education Centre, Agricultural Research Education and Extension Organisation (AREEO), Bandar Abbas, Iran

**Keywords:** Amino Acid, Fatty Acid, Chlorophyta, Phaeophyta, Rhodophyta

## Abstract

Nowadays the exploration and utilisation of food and feed from marine origin is becoming more important with the increase of human population. Macroalgae are rich in nutritious compounds, which can directly be used in human and animal feed industries. The current study presents the screening of chemical components of eight macroalgae species, *Sargassum boveanum, Sirophysalis trinodis, Hypnea caroides, Palisda perforata, Galaxaura rugosa, Caulerpa racemose, Caulerpa sertularioides* and *Bryopsis corticolans* from the Persian Gulf. The results revealed that the eight studied algal species possess high protein (14.46% to 38.20%), lipid (1.27% to 9.13%) and ash (15.50% to 49.14%) contents. The fatty acids and amino acids profile showed the presence of essential fatty acids and amino acids with high nutritional value. Phaeophyta species, *S. boveanum* and *S. trinodis*, showed the highest value of ash content and polyunsaturated fatty acids while Chlorophyta species, *C. racemose, C. sertularioides* and *B. corticolans*, showed the highest level of lipid and protein contents. Rhodophyta species, *G. rugosa* and *P. perforata*, showed the highest essential amino acid content. In conclusion, this study demonstrates the potential of the studied marine species as a nutritional source for human and animal uses.

**Highlights**

- *S. boveanum* and *S. trinodis*, Phaeophyta algal species, showed the highest value of ash and polyunsaturated fatty acids contents.
- Chlorophyta species "*C. racemose, C. sertularioides* and *B. corticolans*" showed the highest level of lipid and protein contents
- *G. rugosa* and *P. perforata* as a Rhodophyta species showed the highest essential amino acid contents

## INTRODUCTION

Fatty acids (FAs) and amino acids (AAs) are important nutritious substances and metabolites in living organism (Chem & Chung 2002). Sixty-eight percent (68%) of all people die from degenerative diseases, i.e. cardiovascular diseases (43.8%), cancer (22.4%) and diabetes (1.8%), which are related to inappropriate FA consumption (Salem *et al.* 1996; Terry *et al.* 2001). Some studies have recognised the vital role of conjugated FAs as bioactive molecules in the treatment of tumors and other cancer-related problems, with varying degree of cytotoxic effects on the cancer cells (Kawagishi *et al.* 2002). The two main polyunsaturated fatty acid (PUFA) classes, n-3 (omega-3) and n-6 (omega-6), play an important role in the prevention of cardiovascular diseases, osteoarthritis and diabetes. It is important to maintain an appropriate balance of omega-3 and omega-6 in the diet (Hibbeln *et al*. 2006; Miyake *et al*. 2010). PUFAs are essential nutrients which cannot or only to a limited extent be synthesised by mammals, so they must be ingested via dietary sources (Broadhurst *et al*. 2000).

Protein is one of the expensive macro-nutrients in ecologic and economic terms and therefore the one requiring the most attention with respect to sustainability (Swanson *et al*. 2013). Proteins provide nitrogen and carbon for the synthesis of gluconeogenesis and energy. Proteins are composed of different AAs and hence the content, proportion and availability of protein's amino acids have been affected in protein nutritional quality. Essential amino acids (EAA) including arginine, histidine, isoleucine, leucine, lysine, methionine, phenylalanine, threonine, tryptophan and valine cannot be synthesised by mammals, so they must be ingested from their diet.

Global nutritional security concerns have been raised in relation to the increasing human population. Consequently, a quest to explore and utilise foods from unconventional sources of both terrestrial and marine origins has been made (Dawczynski *et al*. 2007).

Marine macroalgae consist of more than thousands of species and represent a significant part of the littoral biomass. Macroalgae are classified as brown (Phaeophyta), red (Rhodophyta) and green algae (Chlorophyta) depending on their chemical composition and pigments (Dawczynski *et al*. 2007). Macroalgae are rich in the quantity of nutritious compounds, such as proteins, amino acids, carbohydrates, lipids, fatty acids, vitamins, pigments, minerals and they can be used directly in human nutrition (Aguilera-Morales *et al*. 2005, Ortiz *et al*. 2006, Cardozo *et al*. 2007). The amino acid and fatty acid composition of macroalgae have a wide application in human and animal feed nutrition industries.

Approximately 350 species of marine macroalgae have been reported from the coastline of the Persian Gulf and Oman Sea (Sohrabipour & Rabiei 1996, 1999). Only a limited number of them have been investigated for their chemical composition (Rohani-Ghadikolaei & Abdulalian 2012; Tabarsa *et al*. 2012; Pirian *et al*. 2016; 2017). In the light of the presence of the essential nutritional components for human diet in algae, algae have been recommended as valuable sources of nutritional compounds that may have food applications. The current study presents the screening of chemical components, especially AA and FA contents, of eight species of macroalgae from the Persian Gulf. The aim of this study was to evaluate the nutritional value of the macroalgal species as a source of human complementary food for the management of nutrition deficiencies.

## MATERIALS AND METHODS

### Sampling

**Figure 1:**
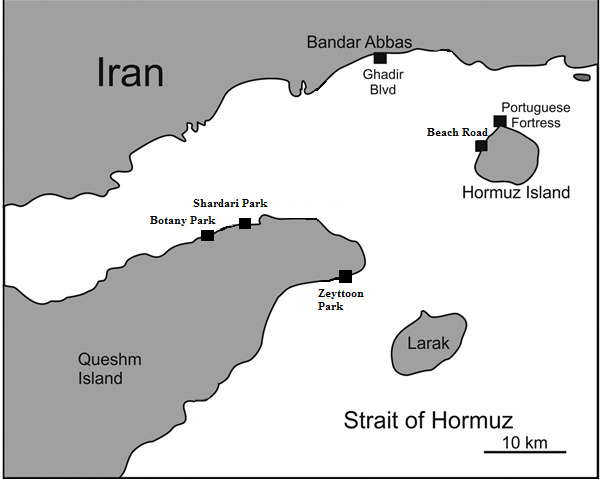
Map of sampling localities in the Persian Gulf, Iran. The sample details are shown in Table 1.

Bandar Abbas, Hormuz and Qeshm Islands were selected as the algal sampling sites. They are located close to each other in the Strait of Hormuz in the Persian Gulf and have similar oceanographic conditions with temperature 18°C–20°C, salinity 39–40 PSU and pH 7.8–8.0 during the sampling period from 15 January 2017 to 3 March 2017 (Table 1, Fig. 1).

**Figure 2:**
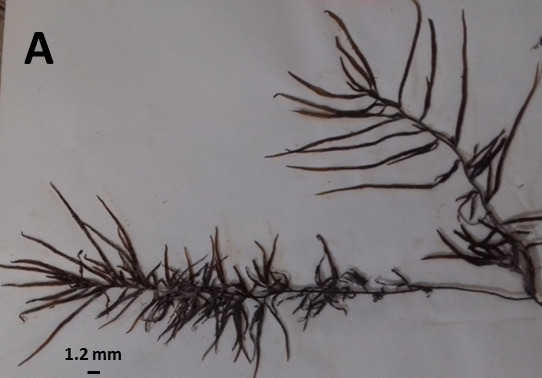

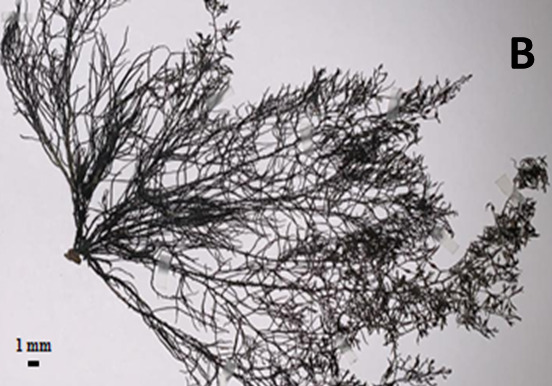

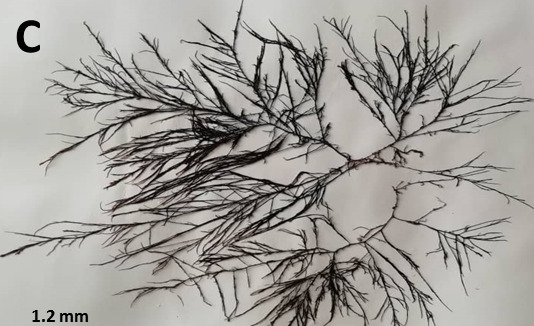

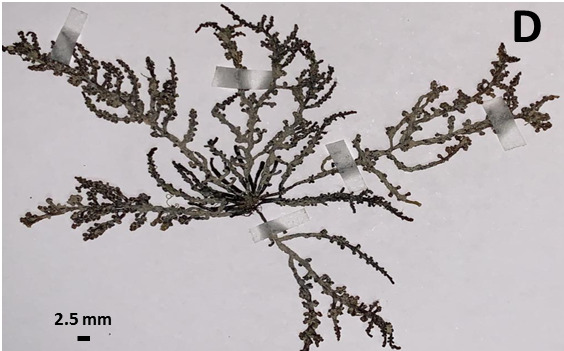

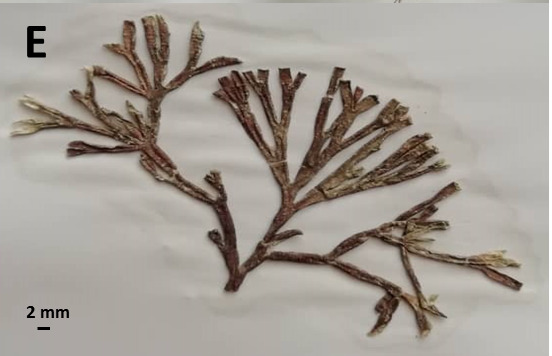

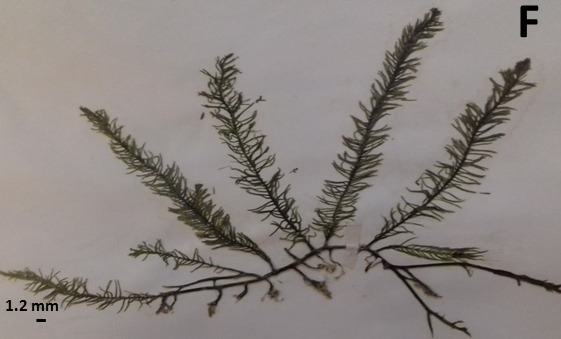

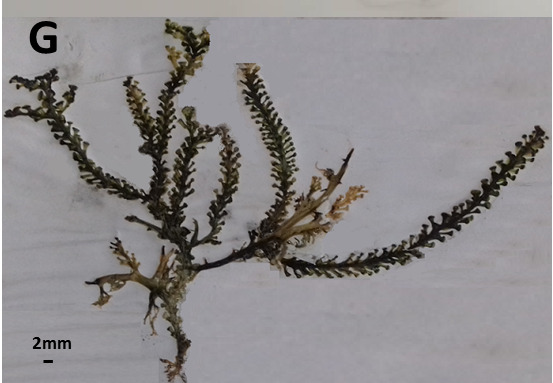

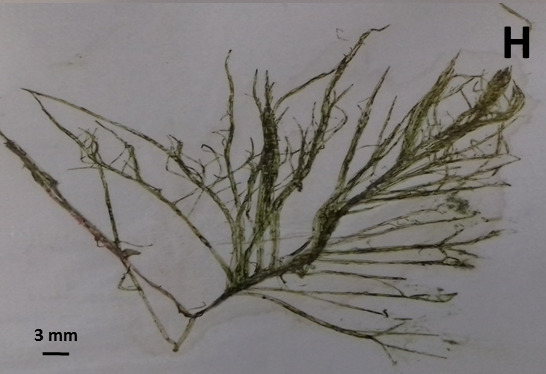
Herbarium specimens of algal samples from the Persian Gulf, Iran. (A) *Sargassum boveanum*; (B) *Sirophysalis trinodis*; (C) *Hypnea caroides*; (D) *Palisda perforata*; (E) *Galaxaura rugosa*; (F) *Caulerpa sertularioides*; (G) *Caulerpa racemose*; (H) *Beriopsis corticolans*.

Algal samples were handpicked from the sandy and rocky seashores of the intertidal zone, first identified in the field, and then transferred to the laboratory for phytochemical analyses. In the laboratory, the samples were washed thoroughly in seawater to remove debris and epiphytes, and prior to further analyses were rinsed with distilled water. After washing, a part of each sample was separated for a part of detailed morphological identification and the rest of the sample was air-dried in freeze dryer and preserved for further analysis in air-tight plastic bags in desiccators at room temperature (25°C). Identification of the tested algae was carried out with standard keys by Gabrielson *et al*. (2006) and Sohrabipour and Rabiei (1996, 1999). Herbarium specimens were deposited at the Algal Herbarium of the Hormozgan Agricultural and Natural Resource Research and Education Centre, Bandar Abbas, Iran. Fig. 2 showed the herbarium specimens of the all eight identified algal samples from the Persian Gulf. Two samples of each of the eight species were processed further chemical analyses.

### Total Lipid Content

The total lipid content of the samples was extracted according to Folch *et al*. (1956). Briefly, freeze-dried algal powder was placed into a glass vial and chloroform: methanol (2:1v/v) mixture was added into it. The mixture was heated at 60°C for 1 h, followed by a filtration step (Whatman GF/A filter) to remove particles. The filtered crude extract was washed with 0.9% NaCl solution, mixed with a vortex spin and let to stand for several minutes until an upper and lower phase established. The upper phase was removed and the lower one, containing the lipids, was evaporated under a gentle stream of nitrogen. The lipid extract was then weighed and expressed as g of total lipids per 100 g dry weight of the sample.

### Total Protein Content

The total protein content of the samples was estimated by using the method by Bradford (1976). The protein contents of the algae were measured by the absorbance (595 nm) and different concentrations of bovine serum albumin (BSA) was prepared as a standard. Finally, the protein contents of the samples were estimated based on the BSA curve and expressed in percentage of dry weight.

### Ash Content

Ash contents of the samples were determined according to the method described by Association of Official Analytical Chemists, AOAC (1995). Briefly, 5 g of a dried algal sample was kept at 525°C for 6 h in blast furnace and weighed. The ash content was expressed as g of ash obtained per 100 g of dry weight sample.

### Fatty Acid

The fatty acid (FA) composition was analysed with Gas Chromatography–Flame Ionisation Detector (GC-FID) after the preparation of fatty acid methyl esters (FAMEs) according to the method by Miller and Berger (1985) with some modifications. Initially for saponification process, the starting solution, containing the sample and NaOH (1.2 mol L^−1^) in an aqueous methanol (50%), was boiled for 30 min. For the methylation process, the sample was acidified with HCl (10 mol L^−1^) and methanolic BCL3 (12%) (catalyst) was added and heated for 10 min. Hexane/diethylether (1:1) was added to the sample for the extraction of the FAMEs. Finally, NaOH (0.3 mol L^−1^) was added to the organic extract (FAMEs). The FAME phase was transferred and evaporated completely using nitrogen flushing. The samples could be stored at −20°C for several weeks. FAME samples were analysed using a gas chromatograph (Varian, 3800) equipped with a fused silica capillary column BPX 70 (25 m × 0.32 mm, film thickness 0.25 μm) and a flame ionisation detector. The run was carried out through a temperature gradient of 160°C–230°C, with an increase rate of 1.5°C min^−1^. FA identification was performed using external standards (SUPELCO F.A.M.E. Mix C4-C24). FA composition was calculated from the total identified FA area and the values were averages of at least three injections of each sample.

### Amino Acids

For amino acid (AA) analyses, triplicate sub-samples were hydrolysed with hydrochloric acid (6 N) in evacuated sealed tubes for 24 h at 110°C. The hydrolysed samples were then analysed in high performance liquid chromatography [HPLC] (Knauer-Germany) Amino Acid Analysis System (Column: C18, Detector: Knauer rf-530, UV Absorbance Detector). Sulphur AAs get partially or completely destroyed during acid hydrolysis. Therefore, cysteine and methionine were first oxidised and then transformed to cysteic acid and methionine sulphone, determined after acid hydrolysis. A set of amino acid standards (Sigma chemicals) was analysed with each set of experimental samples. The identification of the amino acid in the sample was carried out by comparison with retention times of the standards.

### Statistical Analysis

The measuring of chemical contents of all algal samples was carried out in triplicate sub samples, and the experimental results were expressed as means with ± standard deviation (SD). The means of all factors were studied by one-way analysis of variance (one-way ANOVA) by using the SPSS 18 (SPSS Inc., Chicago). Then the individual means were compared using Duncan's post-hoc test. Finally, the results were considered as significantly different if *P* values were less than 0.05 (*P* < 0.05).

## RESULTS

### Total Lipid, Protein and Ash Content

The total lipid, protein and ash contents of eight studied algal species are shown in Table 2. Statistical differences were found between the lipid content of the algal species (*P* < 0.05, Table 2). The total lipid content varied between 1.27% and 9.13% d.w., depending on the algal species. The highest and the lowest lipid content were observed in *Caulerpa sertularioides* (9.13% d.w.) and *Sirophysalis trinodis* (1.27% d.w.), respectively*.* The high value of lipid content in our study was shown in the studied green algal species (*C. sertularioides*, *Bryopsis corticolans* and *C. racemose*)(9.13%–6.12% d.w.).

The protein content of studied algal species (38.20%–14.64% d.w.) showed significant differences between species (*P* < 0.05, Table 2). The *B. corticolans* showed the highest protein content (38.20% d.w.) while *S. trinodis* and *Galaxaura rugosa* showed the lowest protein content (14.64% and 17.82% d.w., respectively) among the studied species (Table 2). The high value of protein content (35.06%–38.20% d.w.) was observed in two green algal species (*B. corticolans* and *C. sertularioides*). The studied algal species showed significant variation in ash content (*P* < 0.05, Table 2). The ash content of studied algal species varied between 15.50 to 49.14% d.w. The highest ash content was observed in *Sargassum boveanum* (49.14% d.w.) and the lowest ash content was observed in *Hypnea caroides* (15.50% d.w.) among algal species (Table 2).

### FA Content

The profile and content of the FAs in the studied algal species are shown in Table 3. The saturated fatty acids (SFA) in the studied species ranged from 142.9 mg g^−1^ FAME in *S. boveanum* to 427.9 mg g^−1^ FAME in *C. racemosa* (Table 3). Palmitic acid (C16:0) was the most abundant SFA and its highest content was observed in green algae; *C. racemosa, C. sertularioides* and *B. corticolans* (342.8 to 361.6 mg g^−1^ FAME) and the lowest in *S. boveanum* (73.4 mg g^−1^ FAME) (*P* < 0.05). The total contents of monounsaturated fatty acids (MUFAs) varied from 282.4 mg g^−1^ FAME in *H. caroides* to 350.7 mg g^−1^ FAME in *C. racemosa* (*P* < 0.05). PUFAs ranged from 212.1 to 530.2 mg g^−1^ FAME. *S. trinodis* and *S. boveanum* showed the highest PUFA content (553.2 to 536.7 mg g^−1^ FAME), while *B. corticolans* showed the lowest PUFA content (212.1 mg g^−1^ FAME). There were significant differences between species in their MUFA and PUFA contents (*P* < 0.05) (Table 3). Oleic acid (C18:1) was the most abundant unsaturated fatty acid which showed the ranged from 193.2 mg g^−1^ FAME in *S. boveanum* to 277.2 mg g^−1^ FAME in *B. corticolans*. Arachidonic acid (C20:4, AA), as the most abundant PUFA, varied from 45.9 mg g^−1^ FAME in *B. corticolans* to 155.5 mg g^−1^ FAME in *S. trinodis*. The SFA/UFA ratio showed lower than 1 in all eight algal species (0.16 to 0.80) (Table 3).

### Amino Acids

The total amino acid content (∑AA) of the eight algal species ranged from 492.5 to 800.9 g kg^−1^ protein, the highest content found in *G. rugose* (800.9 g kg^−1^ protein) and the lowest in *C. sertularioides* (492.5 g kg^−1^ protein) (*P* < 0.05) (Table 4). All eight studied algal species contained all essential amino acids (EAA) for humans, i.e. methionine, leucine, threonine, histidine, lysine, valine and phenylalanine, and nine non-essential amino acids (NEAA) in different proportions. The sum of EAAs (∑EAA) ranged from 190.8 g kg^−1^ protein in *C. sertularioides* to 412.9 g kg^−1^ protein in *G. rugosa.* Leucine and phenyl alanine constituted together the biggest part of the EAA fraction (109.8−63.6 and 80.7−36.1 g kg^−1^ protein, respectively) in all species, except of *Palisada perforata* in which leucine and lysine showed together the biggest part of EAAs (109.8 and 91.0 g kg^−1^ protein). Among NEAAs, aspartic and glutamic acids showed the largest part of the NEAA fraction in green and brown algal species (92.1−63.6 g kg^−1^ and 85.4−66.6 g kg^−1^ protein, respectively) whereas arginine and glutamic acids showed the largest part of the NEAA fraction in the red algal species studied (82.5−66.8 and 113.2−64.3 g kg^−1^ protein, respectively) (Table 4).

## DISCUSSION

Algae have been one of the most versatile sources of bioactive compounds and research on their chemical composition has significantly extended in the past three decades (Cardozo *et al*. 2007; O’Sullivan *et al*. 2010).

In this study, green algae showed higher lipid content (6.12%–9.13% d.w.) compared to red and brown algae. This is in agreement with previous studies (see Chakraborty & Santra 2008; Anantharaman *et al*. 2013; Khairy & El-Shafay 2013), which shown that green algae in general have higher lipid contents than red and brown algae. The lipid content of *Caulerpa sertularioides* (9.13% d.w.) was higher than the lipid content of green algae previously reported from the Persian Gulf; *Ulva prolifera* (6.06% d.w.), *U. paschima* (5.36% d.w.), *U. lactuca* (5.2% d.w.) and *Caulerpa sertulariodes* (2.7% d.w.) (Rohani-Ghadikolaei & Abdulalian 2012; Mohammadi *et al*. 2013; Pirian *et al*. 2016; 2018). Similarly, the lipid content of *C. racemosa* and *C*. *sertularioides* were higher than previously reported for other areas in the world; 0.32% and 3.58% d.w. for *Caulerpa taxifolia* in India, 4.4% and 0.9% d.w. for *C. racemosa* in India, 3.6% d.w. for *C. scalpelliformis* in India, 0.86% d.w. for *C. lentillifera* in Thailand (Ratana-arporn & Chirapart 2006; Chakraborty & Bhattacharya 2012; Murugaiyan *et al*. 2012; Kokilam & Vasuki 2013). The lipid content of *S. boveanum* (2.02% d.w.) was lower than reported for other *Sargassum* species [*S. vulgar* (4.15% d.w.), *S. subrepandum* (3.61% d.w.) and *S. wightii* (2.33% d.w.)] but was higher than other *Sargassum* species [*S. muticum* (1.45% d.w.), *S. polycustum* (0.9% d.w.), *S. wightii* (1.4% d.w.)] (Manivannan *et al*. 2008; Abou-El-Wafa *et al*. 2011; Murugaiyan *et al*. 2012; Chakraborty & Bhattacharya 2012; Rodrigues *et al*. 2015; Pirian *et al*. 2017). *Hypnea cavoides* lipid content (3.04% d.w.) was similar to those reported by other authors for *Hypnea* spp. *(H. musciformis* and *H. cervicornis*) (Anantharaman *et al*. 2013; Mohammadi *et al*. 2013). Biodiesel can be produced from green macroalgae with high lipid contents (Hossain *et al*. 2008). Macroalgae with high lipid contents because of their commonly and low harvest costs can be used as economical option for bioenergy.

Quantitative analysis of protein content of the eight species studied showed that the highest protein content was in the green alga *Bryopsis corticolans* and lowest in the brown alga *Sirophysalis trinodis.* Similarly, Chakraborty and Santra (2008) studied some algal species and recorded the highest protein content in the green alga *Lola capillaris* (40.87%) and the lowest in the brown alga *Dictyota ceylanica* (3.33%). Pycke and Faasse (2015) reported higher protein content in the green alga *Ulva* sp. (33.6%) and lower in the brown alga *Fucus vesiculosus* (5%–8%). In general, red and green algae are characterised by higher protein content compared to brown algae (Ibañez & Cifuentes 2013). Protein content varies between genera but also between species of the same genus. Lower protein contents compared to this study have been recorded for *Sargassum subrepandum* (3.2%) (Abou-El-Wafa *et al*. 2011), *Sargassum tenerimum* (12.42%) and *Hypnea valentiae* (8.34%) (Manivannan et al. 2008), *Sargassum coriifolium* (16.07%) (Haque *et al*. 2009), *Hypnea pannosa* (16.31%) and *Hypnea musciformis* (18.64%) (Siddique *et al*. 2013), *Caulerpa taxifolia* (12.44%) (Kokilam & Vasuki 2013), *Sargassum muticum* (16.9%) (Rodrigues *et al*. 2015) and *Caulerpa lentillifera* (12.49%) (Ratana-arporn & Chirapart 2006). Chakraborty and Bhattacharya (2012) recorded similar values to our study in *Caulerpa scalpeliformis* (32.4%) and *Caulerpa racemosa* (24.8%) from the Gulf of Kutch coastline. These variations in the crude protein content of macroalgae can be due to species, season, environmental conditions and the geographic area (Zucchi & Necchi 2001; Ratana-arporn & Chirapart 2006; Stirk *et al.* 2007). The direct relationship of protein percentage in macroalgae with nitrate of the ambient water was reported by several workers (Banerjee *et al*. 2009). Marine algae are high value food because of their high protein content. Our study also revealed considerably high concentration of protein in the studied algal species from the Persian Gulf.

All samples, with the ash content of 15.50% to 49.14%, fall within the wide variation of ash contents reported for macroalgae. The ash content of *S. boveanum* (49.14%) was much higher than that recorded for other species from the Persian Gulf in previous studies (Rohani-Ghadikolaei & Abdulalian 2012; Pirian *et al*. 2016; 2017; 2018). Khairy and El-Shafay (2013) reported higher levels of ash content of algal species during autumn (with low temperature) compared to spring and summer (with high temperature). Therefore, the high ash content found in our study is likely to be explained by the fact that our samples were collected during the low-temperature season. In general, high level of ash is associated with the amount of mineral elements.

FA are important bio-regulators of many cellular processes and they are precursors in the biosynthesis of eicosanoids, which are essential signaling molecules in humans (Gressler *et al*. 2010). Fatty acids play also important roles in algal physiology, so they may be very responsive to species and environmental changes (Sánchez-Machado *et al*. 2004; Ratana-arporn & Chirapart 2006). In our study, algae mainly composed of saturated FA (SFA) and unsaturated FA (UFA) ranged from the C12:0 to C22:4. Despite the differences among the FA concentrations, palmitic acid (C16:0) was the dominant FA in all eight studied algal species. This is similar to previous studies (Gressler *et al*. 2010; Caf *et al*. 2015, Pirian *et al*. 2016; 2017). In general, all our samples contained the essential FA; C18:2(n6) (linoleic acid), C18:3 (n3) (alpha-linolenic acid), C20:4 (n6) (arachidonic acid) and C20:5 (n3) (eicosapentaenoic acid). C18:1 (oleic acid, n9) was the most abundant MUFA in the species analysed. Lower oleic acid content has been recorded for *Ulva lactuca, Taonia atomaria* and *Padina pavonica* (Caf *et al*. 2015), *Ulva rigida* (Satpati & Pal 2011), *Ulva intestinalis*, *Lola capillaris*, *Ulva lactuca*, *Dictyota ceylanica*, *Catenella repens*, *Polysiphonia mollis* (Chakraborty & Santra 2008), but higher content for *Ulva reticulata, Porphyra* sp*., Palmaria* sp*., Gracillaria changgi* (Ratana-arporn & Chirapart 2006), *Rhizoclonium riparium* and *Gelidiella acerosa* (Chakraborty & Santra 2008), than our studied algae. In this study, brown algae exhibited the highest concentrations of PUFA. Our results were in agreement with previous studies in which brown algae showed higher concentrations of PUFA compared to green algae (Dawczynski *et al*. 2007, Caf *et al.* 2015, Pirian *et al*. 2017), and suggest that green algae have a lower potential, compared to the brown algae studied, as a nutritional source of PUFA for human consumption. According to the World Health Organisation (WHO 2008), the ratio of SFA/UFA should be lower than one in the human diet. Ratio values lower than one were observed for all eight studied algae, making these species potential sources for food and feed products, and from the lipid point of view, they can be incorporated in a more balanced diet.

Unlike most plant protein, algal protein is called complete protein as it contains all essential amino acids for humans. The amount of amino acids varied in the studied algal species, and all species contained all essential amino acids (EAA) for humans. The highest amino acid content was found in the red alga *Galaxaura rugosa*. Similar to our results, in most studies red algae showed considerable levels of EAAs (Gressler *et al*. 2010; Tabarsa *et al*. 2012). In our study, leucine and phenyl alanine were the most common EAAs. The limiting amino acid varies depending on the product: lysine in cereals, methionine in legumes, and cysteine in soybean-fermented products (Li *et al*. 2011; Boland *et al*. 2013). The studied algae contained considerable level of lysine, methionine and cysteine amino acids, suggesting that they would be suitable to complement terrestrial plant protein meals in food and feed industries. These results agreed with previous reports for other algal species (Kolb *et al*. 2004; Mata *et al*. 2016).

## CONCLUSION

Our findings suggested that the studied algal species can be used as alternative nutrient sources of mineral, fatty acids, protein and amino acid for human and animal consumption. Two green studied algal species, *C. sertularioides* and *B. corticolans*, can be used as protein and lipid sources. Two brown algal species, *S. boveanum* and *S. trinodis* can be considered as a nutritional source of PUFA for human consumption. In general, the ratio values of lower than one (SFA/UFA) for all eight studied algae, making them healthy sources for food and feed products. Two red algal species, *G. rugosa* and *P. perforata*, would be suitable as a nutritional sources of EAA. Therefore, the Persian Gulf algae have a great potential for economic application and deserve more attention.

## Figures and Tables

**Table 1:** Macroalgal species with collection details, voucher number and habitat. All sites are located in the Persian Gulf, Iran.

Species	Voucher No.	Source	Latitude and longitude	Habitat	Collection Date
Brown algae					
*Sargassum boveanum*	3031	Ghadir Blvd, Bandar Abbas	N 27° 10.88′ E 56° 22.13'	Sandy	March 2017
*Sirophysalis trinodis*	3039	Shahrdari Park, Qeshm Island	N 26° 57.74' E 56° 16.6'	Rocky	February 2017
Red algae *Hypnea caroides*	3045	Portuguese Fortress, Hormuz Island	N 27° 6.08' E 56° 27.10'	Rocky	January 2017
*Palisada perforata*	3041	Botany Park, Qeshm Island	N 26° 58.42′ E 56° 15.30'	Rocky	February 2017
*Galaxaura rugosa*	3038	Beach Road, Hormuz Island	N 27° 5.6′ E 56° 26.77'	Rocky	January 2017
Green algae *Caulerpa racemosa*	3042	Botany Park, Qeshm Island	N 26° 58.42′ E 56° 15.30'	Sandy	March 2017
*Caulerpa sertularioides*	3043	Shahrdari Park, Qeshm Island	N 26° 57.74' E 56° 16.6'	Rocky	February 2017
*Bryopsis corticolans*	3044	Zeyttoon Park, Qeshm Island	N 26° 56.08′ E 56° 16.50'	Sandy	March 2017

**Table 2:** Total lipid, protein and ash contents of the eight different algal species (as % of algal dry weight).

Species	Total lipid	Total protein	Total ash
*Sargassum boveanum*	2.02 ± 0.05^e^	21.33 ± 0.35^f^	49.14 ± 1.00^a^
*Sirophysalis trinodis*	1.27 ± 0.07^f^	14.64 ± 0.20^h^	22.57 ± 0.15^g^
*Hypnea caroides*	3.04 ± 0.05^d^	25.63 ± 0.46^e^	15.50 ± 0.20^h^
*Palisada perforata*	2.12 ± 0.02^e^	32.05 ± 0.78^c^	26.21 ± 0.10^f^
*Galaxaura rugosa*	4.02 ± 0.12^c^	17.82 ± 0.15^g^	38.31 ± 0.57^d^
*Caulerpa racemosa*	6.12 ± 0.10^b^	29.10 ± 0.23^d^	45.34 ± 0.81^b^
*Caulerpa sertularioides*	9.13 ± 0.23^a^	35.06 ± 0.55^b^	41.18 ± 0.43^c^
*Bryopsis corticolans*	6.52 ± 0.15^b^	38.20 ± 0.62^a^	30.17 ± 0.18^e^

*Note*:

a–h Different superscript letters within each column show significant differences between algal species as determined by Duncan’s post-hoc multiple comparison (*P* < 0.05).

**Table 3:** The composition of fatty acid methyl esters in eight different algal species from the Persian Gulf (in mg of fatty acid g^−1^ of total FAME).

Fatty acids	*Sargassum boveanum*	*Sirophysalis trinodis*	*Hypnea caroides*	*Palisada perforata*	*Galaxaura rugosa*	*Caulerpa racemosa*	*Caulerpa sertularioides*	*Bryopsis corticolans*
C12:0	4.3^d^	5.4^c^	3.6^e^	7.3^a^	6.8^b^	3.0^f^	3.6^e^	3.9^e^
C14:0	27.3^d^^e^	22.5^e^	36.9^c^	21.8^e^	31.1^d^	52.8^a^	57.6^a^	48.1^b^
C16:0	73.4^e^	82.5^d^^e^	153.6^b^	98.9^d^	114.6^c^	361.6^a^	342.8^a^	360.3^a^
C18:0	22.5^a^	17.9^b^	11.4^c^	15.8^b^	16.7^b^	1.4^d^	1.5^d^	1.3^d^
C20:0	9.3^a^	7.2^b^	4.5^d^^e^	6.0^c^	5.4^c^^d^	4.9^d^	4.1^e^	3.7^e^
C22:0	6.1^b^	7.8^a^	4.6^c^	5.6^b^	4.4^c^	4.2^c^^d^	4.6^c^	4.0^d^
C16:1	90.4^b^	102.3^a^	79.5^d^	85.1^c^	82.2^c^	37.3^f^	43.0^e^	31.2^f^
C18:1	193.2^e^	214.7^d^	255.9^b^	201.2^d^^e^	214.5^d^	231.6^c^	259.2^b^	277.2^a^
C20:1	19.6^b^	22.6^a^	14.3^c^	21.9^a^	20.0^a^^b^	13.5^c^	14.8^c^	12.4^c^
C18:2	125.7^b^	128.2^a^^b^	133.3^a^	111.1^c^	103.2^d^	42.5^e^	33.8^f^	30.2^f^
C18:3	44.9^a^	39.5^a^^b^	31.4^b^	33.2^b^	30.5^b^^c^	29.6^c^	31.8^b^	30.3^b^^c^
C18:4	42.5^d^	48.2^c^	58.6^a^	44.5^c^^d^	49.5^b^^c^	54.3^a^^b^	59.3^a^	50.8^b^
C20:4	145.8^b^	155.5^a^	140.7^b^	135.8^c^	122.4^d^	62.6^e^	56.5^f^	46.9^g^
C20:5	74.1^b^	85.3 a	52.8^d^	60.1 c	62.2^c^	49.9^d^^e^	40.6^f^	45.9^e^
C22:4	104.6^a^	96.7^b^	9.5^d^	80.9^c^	98.7^b^	8.9^d^	9.6^d^	8.0^d^
∑SFA	142.9^d^	143.3^d^	214.6^b^	155.4^d^	179.0^c^	427.9^a^	414.2^a^	421.3^a^
∑MUFA	303.2^d^	339.6^b^	350.7^a^	308.2^d^	316.7^c^	282.4^e^	317.0^c^	320.8^c^
∑PUFA	536.7^a^	553.2^a^	426.3^b^	466.8^b^	477.4^b^	247.8^c^	231.6^c^	212.1^c^
SFA/UFA	0.17	0.16	0.28	0.19	0.22	0.80	0.75	0.79

*Note*:

a–h different superscript letters within each row show significant differences between eight algal species as determined by Duncan’s post-hoc multiple comparison (*P* < 0.05).

∑*SFA*: sum of saturated fatty acids, ∑MUFA: sum of monounsaturated fatty acids, ∑PUFA: sum of polyunsaturated fatty acids, SFA/UFA: saturated fatty acids to unsaturated fatty acids.

**Table 4:** Amino acid profiles of eight algal species from the Persian Gulf (in g of amino acids kg^−1^ of protein).

Amino acids	*Sargassum boveanum*	*Sirophysalis trinodis*	*Hypnea caroides*	*Palisada perforata*	*Galaxaura rugosa*	*Caulerpa racemosa*	*Caulerpa sertularioides*	*Beriopsis corticolans*
Thr	41.3^b^	37.1^c^	32.7^d^	35.1^c^^d^	55.4^a^	44.2^b^	18.4^e^	33.9^d^
Phe	80.7^b^	63.2^e^	68.5^d^	52.1^f^	86.2^a^	71.2^c^	36.1^g^	70.9^c^
Val	22.7^d^	35.2^c^	23.0^d^	47.3^a^	40.6^b^	33.2^c^	19.8^e^	23.9^d^
Met	30.5^c^	42.9^b^	26.8^d^	28.5^d^	51.2^a^	30.5^c^	26.6^d^	27.7^d^
Leu	72.8^e^	101.1^b^	69.2^e^	109.8^a^	93.9^c^	87.2^d^	63.6^f^	71.7^e^
Lys	21.1^e^	61.2^b^	21.1^e^	91.0^a^	47.7 c	25.0^d^	18.4^f^	21.9^e^
His	9.1^d^	21.2^b^	5.6^f^	16.7^c^	37.9^a^	7.7^e^	7.9^e^	5.8^f^
Tyr	40.2^b^	58.4^a^	32.7^c^^d^	42.3^b^	23.2^e^	31.7^d^	35.0^c^	33.9^c^
Cys	1.2^d^	28.9^a^	7.2^c^	21.1^b^	1.3^d^	8.6^c^	1.0^d^	7.5^c^
Asp	72.8^b^	92.1^a^	24.5^f^	22.1^f^	55.4^e^	73.5^b^	63.6^d^	69.2^c^
Gly	12.8^b^^c^	6.4^d^	10.2^c^	28.9^a^	16.0^b^	11.5^c^	11.2^c^	10.5^c^
Glu	83.6^d^	91.2^c^	64.3^f^	113.2^a^	105.2^b^	85.4^d^	73.0^e^	66.6^f^
Pro	51.22^b^	61.5^a^	35.7^e^	40.3^d^	42.1^d^	47.8^c^	44.7^c^^d^	30.7^f^
Ser	18.0^c^	6.5^f^	14.6^d^	10.5^e^	35.1^a^	20.6^b^	15.7^d^	15.1^d^
Arg	32.5^d^	38.9^c^	66.8^b^	67.3^b^	82.5^a^	15.0^f^	28.3^e^	25.4^e^
Ala	33.5^c^^d^	15.2^f^	35.6^b^	31.2^d^	27.2^e^	48.4^a^	29.2^d^^e^	36.9^b^
∑EAA	278.2^e^	361.9^c^	246.9^f^	380.5^b^	412.9^a^	299.0^d^	190.8^g^	255.8^f^
∑AA	624.3^c^	761.5^b^	538.5^e^	757.4^b^	800.9^a^	641.5^d^	492.5^f^	551.6^e^

*Note*:

a–h superscript letters within each row show significant differences between eight algal species as determined by Duncan’s post-hoc multiple comparison (*P* < 0.05).

∑*AA*: Sum of amino acids, ∑EAA: Sum of essential amino acids.
